# A methodology for the analysis of differential coexpression across the human lifespan

**DOI:** 10.1186/1471-2105-10-306

**Published:** 2009-09-22

**Authors:** Jesse Gillis, Paul Pavlidis

**Affiliations:** 1 Department of Psychiatry, University of British Columbia, Vancouver, Canada; 2 Centre for High-Throughput Biology, University of British Columbia, Vancouver, Canada

## Abstract

**Background:**

Differential coexpression is a change in coexpression between genes that may reflect 'rewiring' of transcriptional networks. It has previously been hypothesized that such changes might be occurring over time in the lifespan of an organism. While both coexpression and differential expression of genes have been previously studied in life stage change or aging, differential coexpression has not. Generalizing differential coexpression analysis to many time points presents a methodological challenge. Here we introduce a method for analyzing changes in coexpression across multiple ordered groups (e.g., over time) and extensively test its validity and usefulness.

**Results:**

Our method is based on the use of the Haar basis set to efficiently represent changes in coexpression at multiple time scales, and thus represents a principled and generalizable extension of the idea of differential coexpression to life stage data. We used published microarray studies categorized by age to test the methodology. We validated the methodology by testing our ability to reconstruct Gene Ontology (GO) categories using our measure of differential coexpression and compared this result to using coexpression alone. Our method allows significant improvement in characterizing these groups of genes. Further, we examine the statistical properties of our measure of differential coexpression and establish that the results are significant both statistically and by an improvement in semantic similarity. In addition, we found that our method finds more significant changes in gene relationships compared to several other methods of expressing temporal relationships between genes, such as coexpression over time.

**Conclusion:**

Differential coexpression over age generates significant and biologically relevant information about the genes producing it. Our Haar basis methodology for determining age-related differential coexpression performs better than other tested methods. The Haar basis set also lends itself to ready interpretation in terms of both evolutionary and physiological mechanisms of aging and can be seen as a natural generalization of two-category differential coexpression.

**Contact**: paul@bioinformatics.ubc.ca

## Background

**Figure 1:**
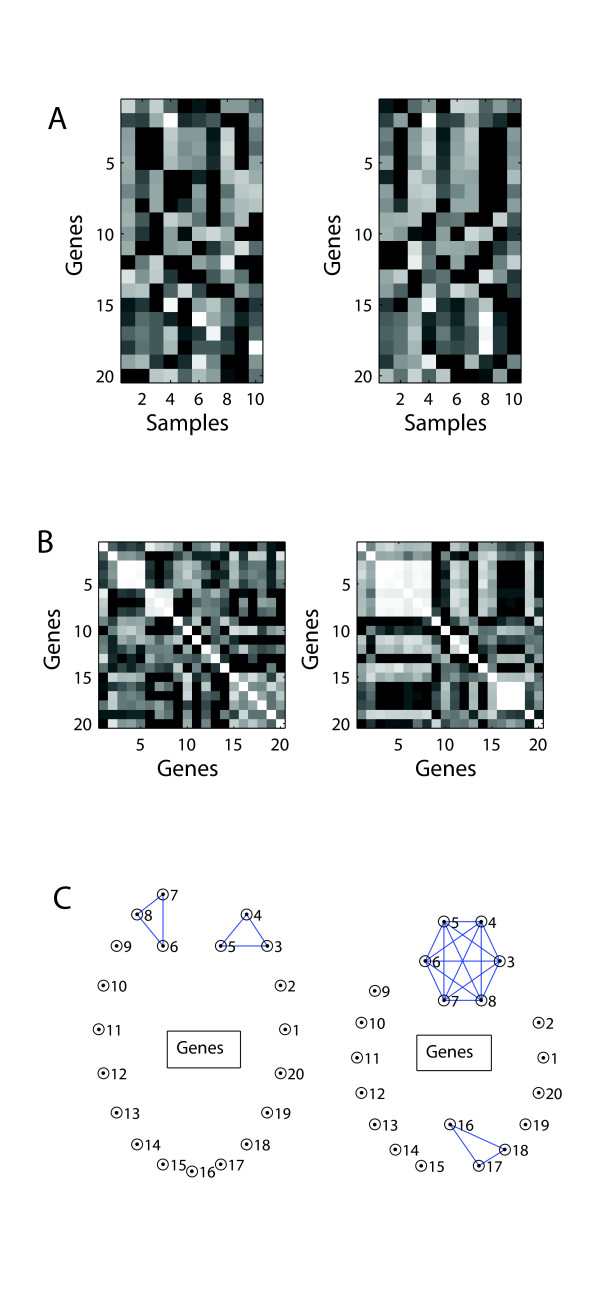
**Schematic of differential coexpression**. The left and right sides of the figure correspond to two hypothetical experimental conditions. A. Heatmap representation of expression levels of 20 genes in 10 samples per condition; lighter shades indicate higher relative expression. The correlations among some genes changes, eg genes 16-18. B. Correlation matrix heatmaps corresponding to the data in A. Light colors indicate higher correlations. The changes in the position and size of the 'blocks' of highly coexpressed genes changes between conditions. C. Coexpression networks generated by thresholding the correlations between each pair of genes, illustrating the concept of 'rewiring'.

Differential coexpression is defined as a change in the correlation relationships between genes. It is a natural extension of the concept of 'guilt by association', which states that functional relationships tend to be reflected in coexpression relationships [1,2]. We think of differential coexpression as potentially revealing 'rewiring' of gene networks, reflecting dynamic changes in the regulatory relationships between genes which can then be 'read out' at the level of transcription. Because of the potential importance of network rewiring, differential coexpression could be useful for uncovering molecular mechanisms of normal processes such as development and aging as well as of disease processes. A schematic outlining the features of differential coexpression is provided in Figure 1.

Differential coexpression has previously been studied primarily in the context of changes in coexpression between two contrasting sample groups such as tumors and normal tissue [3-5]. However, no method to handle multiple ordered groups, such as over age or time, has been proposed.

The current study was motivated by our interest in studying human life stage and aging. For our purposes, we take 'life stage' to include both developmental changes and normal senescent changes. In searching for biomarkers for life stage and aging it has been usual to look for differential expression over time [6-8], sometimes in conjunction with coexpression [9], but not differential coexpression over age. The equivalent task in differential coexpression analysis would look for differences in coexpression across time. Previous expression profiling studies have demonstrated that the expression patterns of age-regulated genes are indicators for a functional measure of aging in humans [10,11]. Because many functional changes occur over the lifespan and biological function often involves the interactions of many genes, we hypothesize that life stage is associated with differential coexpression - many changes in functional relationships or rewiring of transcriptional networks.

To explore these ideas, we used the Gemma database of publicly-available microarray studies [12] for a meta-analysis of expression patterns over age. Using these data we answer three distinct methodological questions:

1) What is an appropriate, generalizable and principled basis set corresponding to differential coexpression over time?

2) How feasible is the repurposing of pre-existing data for a life stage meta-analysis of differential coexpression and how statistically confident can we be in the results?

3) Does differential coexpression provide novel information beyond coexpression or differential expression alone?

In reference to the first question, it is important to consider what a basis set for differential coexpression would constitute. Coexpression over time is taken to be the correlation between genes at each point in time. Differential coexpression, then, is the difference in coexpression over time. If this difference is taken by linear combination of the original data and conforms to other desirable properties (e.g., spans the original data), it will constitute a basis set for coexpression. Because differential coexpression is defined as the change in coexpression, ideally one of the basis set vectors will represent coexpression, while the rest represent differential coexpression. Another important issue is how to partition lifespan into stages that can be compared, and how to compare those stages. One approach to analyzing changes would be to take a derivative of gene coexpression across time, describing the differential coexpression between each age group and the next. However, the derivative comparison will fail to detect gradual changes which can only be characterized over many groups in the long term. Another possible approach to characterize multiple time points would be to compare every age group to every other, but this is highly redundant and ignores the temporal relationship between data points. We hypothesize that changes might occur over both short scales and long scales.

Processes related to aging have been hypothesized to occur over a variety of timescales up to and including linking development and old age [13]. Thus, it may be a desirable property for changes in coexpression over age to be characterized both in their rapid (short scale) change and their gradual (long scale) change.

Thus a good method for differential coexpression should have the following properties:

• It would characterize the change in coexpression at each time.

• It would characterize the change in coexpression over functionally relevant timescales.

• It would form a basis set for the temporal data.

For two groups of data, this reduces to conventional differential coexpression (i.e. a difference between gene correlations).

In the more general case, these three properties suggest we require a transformation incorporating both changes in scale and timing. In order to reduce to conventional two-group differential coexpression, our basis transform of age-specific coexpression data should be seen as taking the difference between groups. This corresponds to the Haar basis set [14], which consists of the difference between adjacent values, adjacent pairs of values, adjacent quartets of values, and so on, in addition to the overall mean. The use of the Haar transform (or D2 wavelet) follows from our belief that different scales of activity will be present. This can be compared to an alternative hypothesis that the relevant timescales are not lifelong, and only consider instantaneous change, or the discrete derivative over time. This too would meet the criteria laid out above (although not the criteria of an orthogonal basis set), under the assumption that functional timescales are not lifelong (each interval can be explained best by looking at the previous interval). We use coarse age bins to validate our approach, but any of the possible basis sets mentioned (Haar, derivative, direct temporal coefficients) can be generalized to any resolution or length of time, and all are particularly appropriate to temporal (or ordered data).

In this paper, in order to validate our choice of the Haar basis as an answer to the first question we posed, we use semantic similarity and statistical independence to show the relative performance of reasonable differential coexpression basis sets. In order to answer the second question posed, we assess the data with respect to a Haar coefficient null distribution and show that repurposing by age produces significant results. Lastly, in answer to our third question, we demonstrate that differential coexpression captures functionally-relevant information not identified using coexpression alone. Because life stage is a process so strongly characterized by changes in function, these techniques developed to characterize life stage may also shed light on how life stage changes function and thus how dysfunction occurs.

## Methods

### Data grouping and Standardization

Human microarray studies from Gemma's database were categorized by their subjects' mean ages into the four groups; "prenatal", "child/young adult" (0-18 years), "adult" (19-54), and "older adult" (55+). Most studies picked fell primarily within one age group. It is important to note that the studies used were not necessarily designed to study age effects, and include a variety of tissues. The selection procedure yielded 3 studies for each age group and 12 in all, encompassing 579 individual microarrays (12 to 193 arrays per study) across a variety of tissues and platforms (Table 1). We analyzed a list of 18534 genes from the UCSC GoldenPath database "known gene" table [15]. Spearman correlations coefficients of the expression profiles between all possible pairs of genes were calculated for each expression study. For genes annotated by Gemma as having multiple probes in a given study, the correlation coefficients for the probes were averaged. For genes sharing a probe (e.g. non-sequence-specific probes), correlations were excluded. Correlation coefficient values were normalized in each dataset to form a Gaussian distribution with standard deviation 1 and mean 0, and averaged across datasets within the same age grouping.

**Table 1:** The Gemma ID number, experiment name, organism part, array design and age category for the experiments are listed in each column.

**Experiments used for analysis**.				
**Gemma ID**	**Name**	**Organism part**	**Array Design**	**Age category**

622	GSE8586	Umbilical cord	GPL570	Prenatal

726	GSE9164	Foreskin cells	GPL5876	Prenatal

233	GSE1397	Brain, heart	GPL96	Prenatal

215	khatua-astrocytoma	Brain	GPL91	Child/young adult

218	pomeroy-embryonal	Brain, kidney	GPL80	Child/young adult

555	GSE5808	Blood cell	GPL96	Child/young adult

585	GSE7586	Placenta	GPL570	Adult

178	GSE80	Muscle	GPL91	Adult

633	GSE8607	Testis	GPL91	Adult

275	GSE4757	Brain	GPL570	Older adult

721	GSE8919	Brain	GPL2700	Older adult

263	GSE5281	Brain	GPL570	Older adult

To allow the investigation of differential expression over age, we computed a relative rank-based measure of expression level for each gene. Each gene's expression level for each study was averaged across samples in each study, converted into a rank with the study, and then averaged within each age group.

### Haar transform

Over our four age groups (prenatal, child/young adult, adult, older adult), the Haar basis consists of four values:

• The averaged correlation across genes across all four time points: (1/2, 1/2, 1/2, 1/2)

• The averaged correlation difference between pre-adult and post-adult: (1/2, 1/2, -1/2, -1/2)

• The averaged correlation difference between prenatal to child/young adult: (1/sqrt(2), -1/sqrt(2), 0, 0)

• The averaged correlation difference between adult to older adult: (0, 0, 1/sqrt(2),-1/sqrt(2))

**Figure 2:**
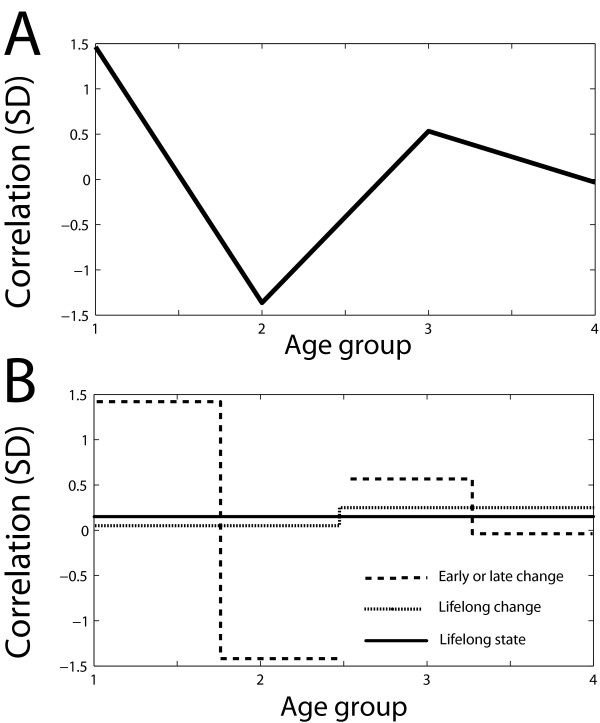
**Method schematic**. A. An example of coexpression data for one pair of genes plotted across age groups. B. The Haar basis set is shown summing to generate the original data points from A. The solid line is the mean value (lifelong state), the dotted line shows the difference between the first half and second half (lifelong change), and the dashed line shows the difference between first and second groups then third and fourth groups (early and late change, respectively). Each successively smaller scale is graphed on top of the sum of previous scales, summing to the original.

The four Haar differential coexpression values are the dot product of these vectors with the age grouped expression data. Note that the first Haar coefficient represents the average coexpression across all ages. The remaining three coefficients represent differential coexpression and no direct aspect of coexpression (since the coefficients are orthogonal). Henceforth "differential coexpression coefficients" excludes the first coefficient. For each age group, the mean coexpression was calculated as a weighted sum of the underlying experiments, where the weighting was the number of microarrays used in the experiment. The variance of temporal coefficient values across the experiments used was calculated. Two other basis sets were also examined which meet our criteria to varying degrees and overlap somewhat with the Haar basis set: The zero-padded discrete derivative and the direct temporal basis set. A method schematic for the Haar transform is shown in Figure 2.

The first coefficient in the discrete derivative remained the first value in time, while the remaining coefficients were calculated by taking a discrete derivative, or difference between successive pairs of values across time. This set will be referred to as the derivative set. Conceptually this overlaps with the direct temporal basis first coefficient and the Haar third and fourth coefficients, but lacks any scale variation, such as that found in the Haar second coefficient.

We performed the Haar transform both on the averaged correlation between pairs of genes (as a time series across our age groups) as well as on the averaged expression level ranks of individual genes. To ensure that the differential coexpression findings may not be explained by simpler underlying changes in expression level, genes exhibiting significant differential expression values at a given time and scale (top 5% rank change) were removed. If a gene exhibited differential expression for one coefficient, other coefficient values for the same gene which were not exhibiting differential expression were retained. This procedure resulted in the removal of 448 genes for each coefficient, on average. (There was slight variation in the number of genes removed due to variation in missing data across experiments). This allows our analysis to focus on genes which are relatively stable in expression level, but which might exhibit changes in coexpression relationships with other genes. In pure biological discovery, as opposed to this validation of the utility of differential coexpression and this methodology specifically, such a step may not be necessary.

### Statistical Assessment

To determine the statistical significance of a differential coexpression pattern and estimate false discovery rates, we need to develop an appropriate null distribution. We treat each Haar coefficient as random variables. Each Haar coefficient is a weighted sum of the correlation values from the underlying experiments. We observe that since a sum of random variables is the convolution of their probability density functions, and the probability density functions of the correlations were normalized to the unit Gaussian (section 2.1), a null distribution is readily calculable as a convolution of Gaussians. This is the null distribution to calculate false discovery rates for coexpression. For differential coexpression the situation is more complex. We are instead asking if the specific data grouping (by age) produces heavier tails in the distribution of coefficients than would occur due to random grouping of the data. Note that this does not require the data to be independent, which, in fact, it will generally not be (since genes are frequently coexpressed in multiple data sets) [2]. The variance for each possible data grouping is calculable from the standard formula for a sum of weighted variables:

(1)

where n is the number of experiments with weighting Ai and variable Xi for indices i, j, and k from 1 to n. The average correlation between experiments, *ρ*, was calculated from the weighted sum in equation 1, excluding i = j terms. The Haar coefficient variance was then calculated from the above by multiplying AiAj terms by -1 where experiment's i and j belong to different age groupings. Thus, with equal weightings, the Haar coefficient variance using the average correlation would be

(2)

A final complication is that there are missing data, as not all data sets have data for all genes (and thus gene pairs). Failure to address this would cause us to underestimate false discovery rates. We therefore computed null distributions specific to the combination of data sets in which any given gene pair was measured. Fortunately the number of combinations which must be considered is small as many combinations are not present (i.e., missing in data sets 1, 5,7 would be one combination, missing only in data set 4 would be another combination, etc). Fewer than 10 combinations of missing data accounted for 95% of all data combinations, although the true total number of combinations of missing data was as high as 88 across all datasets. The methods code is available in the supplementary matlab methods data (see additional file 1: Supplementary.rar) and at .

### Semantic similarity validation

Semantic similarity was used to assess the quality of links generated using different basis sets [16]. The number of overlapping Gene Ontology (GO) terms for each pair of genes was calculated. The change in this semantic similarity was calculated as a function of an increasing threshold on the coefficient score for each of our basis sets; that is, the semantic similarity, y, of the top x% of high value coefficients (representing a large positive change in correlation) was calculated as a function of x. Links were generated across all coefficients for each basis set.

### Gene function prediction validation

All GO groups of genes for which 10-30 genes had differential coexpression values as calculated in section 2.3 were assembled using the Gemma web services (see supplementary data at ). This size range was chosen for computational tractability of cross-validation, and to avoid using GO categories which overlap extensively. This generated 648 separate GO groups with 91% coverage of the genes for which a GO category is assigned.

We used these GO groups for validation of the differential coexpression results. By an analogy with the use of coexpression to predict gene function, we propose that each gene within a given GO set might have a characteristic differential coexpression relationship with genes inside the set and a characteristic relationship with genes outside the set. For each gene inside the set, gene A, the respective distributions of differential coexpression coefficients can be calculated. An arbitrary gene, gene B, outside the set has differential coexpression values with gene A that may also be calculated. We may then ask if gene B's relationship with gene A resembles gene A's relationship with other genes inside the set or with genes outside the set. As a control, the same calculation was performed using the coefficient representing coexpression.

A leave-one-out methodology was employed in which one gene was removed from the GO group to form a new set. Then for each gene now in the set, the rank score it possesses with each other genes in the set was calculated, and the genes outside the set ranked by how close their own score is to the in-set score. This procedure was then repeated by rotating through each gene in the set and each coefficient for differential coexpression. Ranks across coefficients were averaged and values re-ranked. Then, this entire method was repeated by rotating through each gene originally in the GO group (leave-one-out methodology). Receiver operator characteristic (ROC) curves were then calculated from this data.

ErmineJ [17] was used to perform overrepresentation analysis (under the ROC setting) for each gene for each coefficient. The full gene set was used for the overrepresentation analysis (e.g., not limited to 10-30 genes as in the ROC analysis). Multiple test corrected p values less than 0.001 were retained for each gene. ErmineJ uses Benjamini-Hochberg correction [18].

## Results

To test our approach, we analyzed differential coexpression across human lifespan in a corpus of 12 expression studies (579 individual microarrays in total). This produced 4 symmetric matrices of 18534 by 18534 genes with potential Haar coefficient values, consisting of coexpression (the first coefficient) or differential coexpression over different time (the other three coefficients). Discounting missing data, this yields 320,201,152 data points.

To gain preliminary insight into the functional relevance of differentially-coexpressed genes, we applied GO term enrichment analysis. For each gene (a "query"), we ranked all other genes by the extent to which they are coexpressed or differentially coexpressed with the query (based on each of the four Haar coefficients). We selected GO terms which were significantly associated with each ranking, and summarized the results by counting how many times a GO term appeared, summed across all genes (Table 2; full data available in the supplement). The strongest coexpression patterns are given by the first coefficient (first column of Table 2) while differential coexpression patterns are represented by the other columns. For example, the GO term "hormone activity" was very frequently (83% of the time) associated with genes showing changes in coexpression across the first two age groups. In agreement with our hypothesis that different time scales might be associated with changes in different functions, the different time scales give high rankings to different GO terms. A more detailed analysis of the biological relevance of the age-related changes we found with reference to specific genes and pathways will be described elsewhere; here we focus on validating the method we developed.

**Table 2:** For each time period listed, an overrepresentation analysis was performed for each gene's coexpression or change in coexpression.

**The top 5 Gene Ontology (GO) groups for each age range.**			
**Coexpression**	**Lifelong change**	**Early change**	**Late change**

translational elongation (GO:0006414)	Glycolysis (GO:0006096)	hormone activity (GO:0005179)	ATP metabolic process (GO:0046034)

Mitochondrial membrane part (GO:0044455)	aerobic respiration (GO:0009060)	muscle system process (GO:0003012)	ribonucleoside triphosphate metabolic process (GO:0009199)

ribosomal subunit (GO:0033279)	cellular respiration (GO:0045333)	Hemostasis (GO:0007599)	nucleotide biosynthetic process (GO:0009165)

regulation of ubiquitin-protein ligase activity (GO:0051438)	glucose metabolic process (GO:0006006)	secretory granule (GO:0030141)	monovalent inorganic cation transmembrane transporter activity (GO:0015077)

proteasome complex (GO:0000502)	pigment granule (GO:0048770)	calcium ion homeostasis (GO:0055074)	purine ribonucleotide biosynthetic process (GO:0009152)

Corrected p values <0.001 were assembled and the top 5 GO groups across all genes are shown for each category.

In the following sections, we analyze the differential coexpression data set in several other ways. First, we discuss how statistical significance of differential coexpression can be determined. Second, we compared our Haar transformation approach to one based on derivatives of coexpression changes or other coexpression measures. Finally, we explored whether differential coexpression is relevant to gene function using an analysis of Gene Ontology categories.

### Statistical analysis

**Figure 3:**
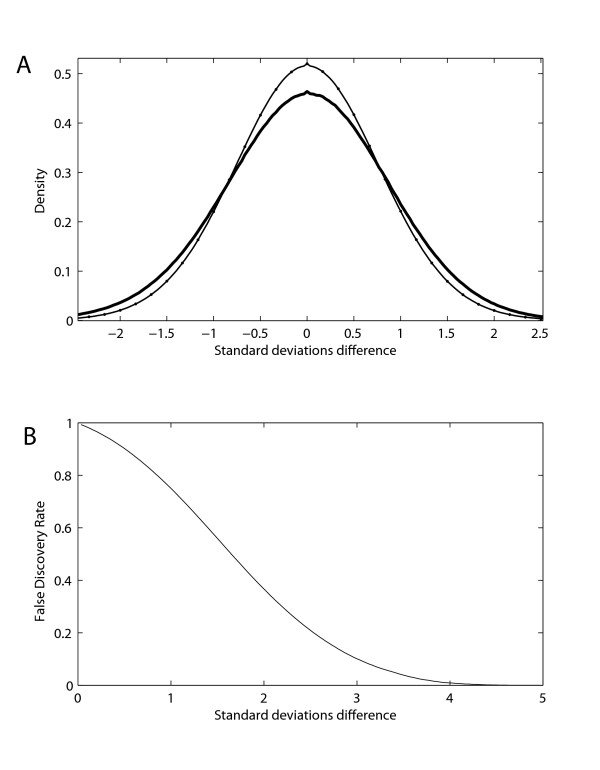
**Statistical properties of Haar coefficients**. A. The inner curve (thin curve) is the probability density function generated from generating differential coexpression coefficients grouping the data cross all possible combinations using data categorized as adult and older adult. The heavier tailed curve (thick curve) is the probability density function resulting from grouping the data by age. The dots are the calculated null distribution for the differential coexpression coefficient. The x axis is the correlation difference between the two groupings of data used. B. The false discovery rate is shown. Again, the x-axis is the difference between coexpression in older adults and coexpression in adults, when each experiment is normalized to a unit Gaussian.

To determine whether the patterns of differential coexpression we observed could occur by chance, we compared the distribution of Haar coefficients found in the age differential coexpression analysis to null distributions computed as described in the methods. If the grouping of data sets by age group is meaningful, the distribution of Haar coefficients should be heavy-tailed compared to the null distribution. To test this we compared the real data to a theoretical null distribution (equation 2) as well as an empirical distribution computed by generating every possible random grouping of data sets (of the same size as in the real experiment), repeating the Haar analysis, and constructing histograms of the values. In Figure 3A, the probability density function across all possible random reassignments of the experiments to adult vs. older adult is shown (inner curve). Experiments were weighted equally for this analysis so that the null distribution would remain the same across data reshuffling. The null distribution calculated from the average correlation equation is shown (dots sitting atop the inner curve), showing an excellent agreement between the analytic prediction and the shuffled data. The distribution of the actual age categorized data is shown (heavy-tailed curve). This is the largest variance distribution of all possible combinations of data (by label reassignment), indicating that grouping data by age reveals significant differential coexpression. While other combinations of data generate heavy tails reflective of other heterogeneities in the data (e.g., tissue), grouping by age produces the strongest effect. False discovery rates can then be calculated, shown in Figure 3B, confirming the presence of significant differential coexpression. As with a coexpression matrix, universal statistical significance would also be problematic, and the sparsity of significance values we see here is similar to that present in coexpression analyses (e.g.,[2,19]). The false discovery rate shown is representative of the average across all weighted null distributions for each Haar coefficient; that is, the tails are similar to those seen in each of the, say, 88 combinations of missing data referred to in section 2.3. Lifelong variation generally produced the heaviest tails with no other discernable trends.

Because this is a large and complex data set, we stress that important trends may exist outside of examining only the most statistically significant cases. Our validation experiments below use all the data; however, we have also constructed an adjacency matrix consisting of the most significant gene-pairs across all coefficients (false discovery rate <0.01). This produced 367161 significant unique gene-pair relationships, available as supplementary data.

### Comparison to other basis sets

As mentioned, an alternative basis is the discrete derivative of coexpression, starting with the first time point's expression level and then each subsequent coefficient reflecting change from the previous value. It might also be reasonable to question whether changes in coexpression are helpful to consider at all, rather than simply independently observing each time point for coexpression. In that case, the natural basis set would be the direct temporal basis set (age groups 1-4) of coexpression data directly. Each pair of genes has 4 coefficients associated with it. There is little point to this if significance in one coefficient implies significance in another.

Supplementary Figure 1 (see additional file 2: supfig1_sup.eps) shows the correlation of basis coefficients with one another within each basis set. Strongly correlated coefficients will diminish the chances of observing any change with age. The lack of orthogonality in the derivative basis set makes it the worst performing set with respect to independence (mean correlation coefficient magnitude of 0.27). By this standard, the Haar basis set performs better (correlation of 0.08) than the derivative basis set. The Haar coefficients are also less correlated than the direct temporal basis set (correlation of 0.16). The corrected Haar basis set performs better still (correlation of 0.03). Just adding noise to our data would produce a similar effect so it is important to verify that this increase in independence does not cause a decrease in functional links.

**Figure 4:**
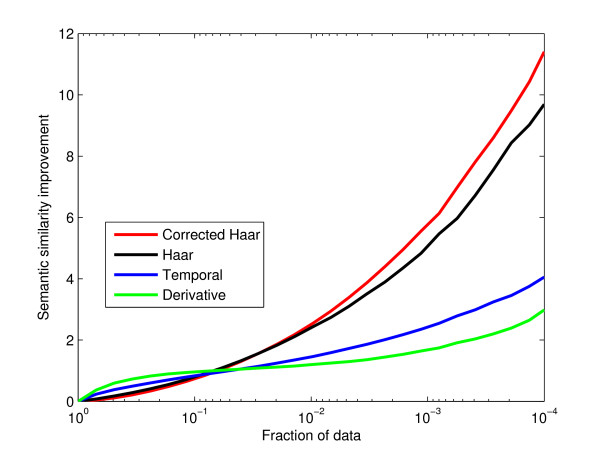
**Semantic similarity of extreme basis coefficients**. Starting from the left, only the more extreme coefficient values are retained. Corrected Haar values (red line) perform the best, followed by Haar values (black line), age segregated values (blue line), and last derivative basis set values (green line)

Figure 4 shows the change in semantic similarity at increasingly stringent cutoffs for selecting gene pairs. A link persists and is included in the semantic similarity if it passes the changing threshold in any coefficient. Starting from the left (all data included) and moving to more positive thresholds, up to the top 0.01% of data, semantic similarity rises for all measures. The reversal in performance is due to the good performance of extremely negative values (also statistically significant). The Haar basis set performs very well (thin solid line in Figure 4) with the lifelong differential coexpression being its highest performing coefficient. In this case, the improvement is a substantial fraction of the average gene's maximum improvement possible, calculated by averaging the maximum semantic similarity pairing for each gene and then averaging across genes to give an improvement of 18.4. The shorter timescales perform less well in differential coexpression, as also seen in the weaker performance of the derivative basis set (Figure 4, dotted line). The derivative basis set also suffers here due to its lack of orthogonality, performing even worse than the age segregated values (direct temporal basis set; dashed line). Correcting for missing data in the Haar basis set improves performance, as expected (thick line).

### Gene function is reflected in differential coexpression patterns

**Figure 5:**
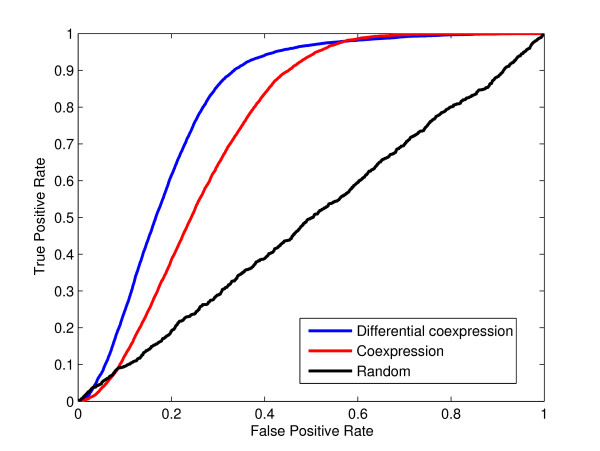
**Functional categories of genes can be predicted by differential coexpression**. The blue line curve shows the result of leave one out validation to generate an ROC curve for reconstructing GO categories containing 10-30 genes using their differential coexpression values with AUC 0.81. The red line shows if coexpression values alone are used (0.74). The black line curve shows the resulting ROC curve if in place of GO sets, random sets with 10-30 genes are used (AUC 0.49).

To evaluate the functional significance of differential coexpression in age, we used annotations provided by the Gene Ontology [20]. We used a cross-validation approach (see methods) to test whether gene function can be predicted on the basis of differential coexpression, as compared to the ability to do so using coexpression alone. Results are shown in Figure 5. For each training set (a GO group minus one in-group gene, rotated through each in-group gene) classification of a testing set (all genes outside the GO group plus one in-group gene, rotated through each in-group gene) was performed. Note that genes exhibiting strong differential expression across age were removed to ensure that changes in coexpression are not better explained by differential expression of the same genes [21]. The area under the curve (AUC) for the differential coexpression ROC curve is 0.81 with a standard deviation 0.09 across the GO groups. The AUC of 0.81 represents the probability of correctly assigning a higher score to a random in-group gene over a random out-group gene (out of one of each). As a control we also attempted this classification using coexpression alone and obtained an AUC of 0.74 (Figure 5). Random sets of genes (of the same size) were used to generate the expected identity line with an AUC of ~0.5 (Figure 5). Using coexpression in conjunction with differential coexpression does improve performance further, although slightly (AUC of 0.84), suggesting that coexpression and differential coexpression are somewhat independent, as expected.

## Discussion

Our results suggest that the Haar basis set view of differential coexpression is a useful tool for capturing functional relationships between genes as their expression changes over ordered sets of conditions. Using this method, we provided evidence that there is substantial differential coexpression associated with life stage. In addition to their good performance compared to a derivative approach, the Haar basis had an additional feature making it attractive for meta-analysis, in that individual studies with samples containing multiple 'ages' could be combined and analyzed at multiple scales. For example, if a study covered the age groups prenatal and child/young adult, it could be included in both, ensuring it contributed to only the first and second Haar coefficients, as appropriate for the scale at which the study was performed. It should be noted that since its introduction, the Haar transform has become more widely known as the first wavelet transform. Our use of the wavelet transform is unconventional because we have only four time points, in contrast to more typical applications where temporal resolution is much finer. However, in this case, the specific wavelet transformation is a useful basis set with a biological underpinning, and having a convenient generalization. Thus the wavelet method could equally well be applied to studies specifically geared toward the analysis of data with different or finer temporal resolution, or to other ordered conditions.

### Differential coexpression over time using the Haar basis

The relatively poor performance of the temporal coefficient method was not surprising, since lifelong coexpression is not an independent coefficient in this basis set and will dominate at each time point. That is, if a gene pair is highly coexpressed, even if it is also highly variable over age (yielding large coefficients in the other two bases), it is still quite likely to be considered highly coexpressed at all ages. The inferior performance of the derivative basis suggests that longer scale dynamics are relevant to expression changes with age. This would seem consistent with the proposal of Barker et al. [22] that fetal programming can play a significant role in determining factors affecting longevity [23].

One possible concern with our study is that we have mixed data sets from various tissues. The precise tissue for prenatal vs. adult, for example, may differ. Likewise there are study parameters (such as population type or array design) that might vary with age. It could then be argued that the differential coexpression across age that we see is simply a proxy measure for differential coexpression between tissue type. However, this is not consistent with the statistical assessment we have performed. It is quite likely that controlling for tissue (and other experimental parameters) would yield even better results, but even absent that, grouping by age produces a clear statistical effect compared to the null distribution, relative to randomized groupings compared to the null distribution. Previous studies (e.g., [6]) have shown tissue dependent aging signatures which may well be excluded from the present analysis. However, we find differential coexpression across tissue to have a less pronounced signature than across aging. To bolster this finding, we examined the tissue dependent coexpression signature in a wholly independent dataset, the full mouse experiment sets available in Gemma (492 datasets). The coexpression signatures in all brain datasets is very similar to the coexpression in all datasets excluding brain tissue using Gemma's significant coexpression list [12]; in particular, the no-brain set (sparsity of 2.5%) obtains 98% of the significantly coexpressed gene pairs in the brain only set of significantly coexpressed gene pairs. This suggests there is a common coexpression signature which may be differentially present over age, but does not imply there is not differential coexpression between tissues. A particular concern might be that tissue heterogeneities particularly could have an effect on the last of our Haar coefficients (all brain data), but this does not appear to be the case. Comparing brain to no-brain data across all datasets produced false discovery rates much higher than across aging, suggesting it is not a significant contributory factor in this case, where brain (widespread expression) is mixed with a variety of tissues without strong homogeneities. Another consideration is that because differentially expressed genes are easily differentially coexpressed, our filtering by differential expression removed prominent cases of differential coexpression, and may well have contributed to minimizing tissue dependencies in our analyses. However, those trends are better considered as cases of differential expression (a much more tractable phenomenon) than differential coexpression.

Another advantage of the Haar method is that studies originally performed over a variety of ages can be more effectively repurposed since including a study across multiple ages tends to remove its effects not operating at the scale of the original data (that is, including a study in older adult and adult will tend to have no late-in-life only differential coexpression effects).

Our results make the point that in general, the choice of an appropriate basis set is important to extracting information from the data. A contrasting approach would be to choose a basis *post hoc*, as is effectively done in any dimension reduction. This presents a number of problems. First, it will vary from dataset to dataset and introduce new normalization difficulties. The variation from dataset to dataset would also reduce the general applicability of any results since any past findings would have to be reinterpreted in the context of whatever basis set is calculated for the new work. In addition, there would be no reason to see the new basis set as a form of differential coexpression precisely since it is unlikely that a single component would end up wholly representing coexpression (thereby removing it from the others, as was the case with the Haar basis). Finally, a principled transformation of the data can be geared to offer value as an interpretive tool, as opposed to a purely methodological tool.

A reasonable concern for our statistical analysis might be that in considering only consistency of rank across experiments, we have thrown away useful information in the form of the correlation distributions themselves. In that case, a natural alternative to the non-parametric statistical analysis we have performed would be to consider the distribution of correlation values themselves, and then combine those p values and determine the false discovery rate. This could be performed by using Fisher's transformation [24] to test the significance of correlation values and then combining datasets using a meta-analysis technique such as Fisher's method [25]. Such an analysis would be erroneous for a number of reasons. Most importantly, it would be dominated by heterogeneity unrelated to the variable of interest because significance in any one experiment will tend to dominate (since usually very significantly heavy-tailed), but individual experiments have many heterogeneities unrelated to age. More reasonably, one could construct null distributions by computing a reasonable sample of distributions with randomly labeled datasets. As we show, in figure 5, this is quite close to the approach we took (absent weighting), but would become very cumbersome for even intermediate numbers of datasets (remembering that each combination of missing data requires a different null distribution).

### Biological interpretation of the Haar approach

The GO group overrepresentation analysis (Table 2), while providing only a high-level coarse overview, suggests some possible interpretations of differential coexpression with age. It is biologically plausible that static coexpression (the first column in Table 2) is dominated by GO terms reflecting stoichiometric complexes such as protein synthetic machinery. In contrast, we expected more dynamic processes to show differential coexpression. It is therefore interesting that "Hormone activity" shows strong early life differential coexpression. Similarly, more gradual ("lifelong", second column of Table 2) differential coexpression should reflect processes implicated in gradual aging. Metabolic processes, such as glycolysis are interesting since they are implicated in ageing diseases over a long temporal scale (e.g., [26]). Table 3 shows the top three most significant differential coexpression relationships for each coefficient along with KEGG pathway information [27] and gene age information from the GenAge database [28]. The gene pairs shown in common KEGG pathways also have significantly high semantic similarity (>99%), and the list is overrepresented with respect to the GenAge gene list of 261 human aging associated genes (p < 0.01). Biasing from experimental heterogeneity was not evident among these other top 10 choices. For example, while Alzheimer's related data is present among the experiments, it was not among those contributing to the early differential coexpression coefficient. More detailed exploration of these results is a topic for future study.

**Table 3:** The three gene pairs exhibiting the largest change in correlation with one another over lifespan for each differential coexpression coefficient are shown.

**Genes differentially coexpressed over age**.			
**Lifestage**	**Gene 1**	**Gene 2**	**KEGG relationships**

Lifelong change (+)	platelet-derived growth factor receptor, beta polypeptide (PDGFRB) *	SHC (Src homology 2 domain containing) transforming protein 1 (SHC1)*	Focal adhesion (hsa04510) Glioma (hsa05214)

Lifelong change (-)	prostaglandin E receptor 3 (subtype EP3) (PTGER3)	abl-interactor 1 (ABI1)	

Lifelong change (+)	hematopoietic cell-specific Lyn substrate 1 (HCLS1)	CD14 molecule (CD14)*	Pathogenic Escherichia coli infection (hsa05130)

Early life change (-)	eukaryotic translation elongation factor 1 alpha 1 (EEF1A1)*	exportin 5 (XPO5)	

Early life change (-)	glutamate receptor, metabotropic 4 (GRM4)	calcium channel, voltage-dependent, P/Q type, alpha 1A subunit (CACNA1A)*	Taste transduction (hsa04742)

Early life change (-)	'6-pyruvoyltetrahydropterin synthase (PTS)*	thioredoxin domain containing 9 (TXNDC9)	

Late life change (+)	Janus kinase 1 (a protein tyrosine kinase) (JAK1)	mitogen-activated protein kinase 9 (MAPK9)*	Pancreatic cancer (hsa05212)

Late life change (-)	progesterone receptor membrane component 1 (PGRMC1)	ubiquitin-conjugating enzyme E2N (UBC13 homolog, yeast) (UBE2N)	

Late life change (-)	neutral sphingomyelinase (N-SMase) activation associated factor (NSMAF)	zinc finger protein 609 (ZNF609)	

The timing and scale of change is listed in the first column, along with the direction of correlation change. The pairs of genes are listed in the second and third column, with asterisks to indicate membership in the GenAge database. The last column indicates if the genes are present in a common pathway from the KEGG database.

Our data covers a range of age categories including both development and senescence. Two fundamental theories of senescence are Williams [29] theory of antagonistic pleiotropy and Medawar's [30] theory of mutation accumulation. These theories present an interesting interpretation in the context of our differential coexpression coefficients. Antagonistic pleiotropy posits a long-scale connection between early states and late states, in which a characteristic useful in youth is harmful later (e.g., [31]). A changing functional role over age in this way would be a good candidate for finding differential coexpression, and, in particular, we would expect such differential coexpression to be present in the coefficient at the appropriate time-scale. We would expect to see antagonistic pleiotropy candidate genes showing significant values in their lifelong change coefficient (youthful tradeoff), while mutation accumulation should particularly exhibit enrichment significance in the fourth coefficient (senescent change). The third Haar coefficient maps most readily onto developmental change, representing rapid and early changes in coexpression which we are tempted to interpret as developmentally-related changes in function.

More specific mechanistic interpretations of function and dysfunction over age also map more readily onto a Haar basis than other bases because they typically involve both a factor in time and scale. As previously mentioned, Barker's theory of fetal programming suggests a long term effect between early and late. One well studied mechanism reviewed by Maric [32] involves fetal programming for high blood pressure. While some research has cast doubt on the importance of fetal programming in longevity itself [33], there may well be similar processes that do not affect longevity simply, but do affect function in more complex ways [34] or relate developmental and late stage changes [35]. Because the Haar coefficients can capture both the scale and timing of this event, they might serve to elucidate the unknown genetic causes for the well characterized physiological changes. With only four groupings by age, interpretation of this sort in our data must remain somewhat restrained, but finer resolution age groupings could make this a valuable characteristic of our method.

## Conclusion

Differential coexpression over age generates significant information about the genes producing it. Our Haar basis methodology for determining age-related differential coexpression performs better than either a derivative based method, or using the age groups independently. The Haar basis set also lends itself to ready interpretation in terms of both evolutionary and physiological mechanisms of aging and can be seen as a natural generalization of two-category differential coexpression. The good performance across the multiple GO sets implies that age related differential coexpression may be a common process due to the degree to which life stage produces changes in function and functional relationships. Because our Haar-based method for differential coexpression draws upon such a well established signal processing tool for temporal data, it offers a well characterized, efficient and convenient avenue for further study.

## Authors' contributions

PP and JG conceived the study. JG performed all the experiments. PP and JG prepared the manuscript. PP provided project oversight. Both authors read and approved the final manuscript.

## Supplementary Material

Additional file 1 **Supplementary matlab methods data**. The matlab m-files to perform differential coexpression and data files for figure generation. Click here for file

Additional file 2 **Supplementary figure 1**. The correlation between coefficients for each basis set is shown. Click here for file
